# Time of course CD64, a leukocyte activation marker, during extracorporeal circulation

**DOI:** 10.1186/cc10621

**Published:** 2012-03-20

**Authors:** S Djebara, P Biston, F Emmanuel, A Daper, M Joris, P Cauchie, M Piagnerelli

**Affiliations:** 1 CHU Charleroi, Belgium

## Introduction

CD64 is a high-affinity leukocyte receptor for the Fc portion of IgG [1]. As CD64 expression on neutrophil cells (PMNs) is upregulated specifically after bacterial stimulation, it could be used to discriminate inflammatory states from bacterial infections [1-3]. The objective was a comparison of the time course of CD64 expression on PMN cells between patients undergoing cardiac surgery with extracorporeal circulation (ECC) with septic patients.

## Methods

Prospective study realized in the ICU of CHU Charleroi (Belgium). Thirty-nine patients scheduled for a cardiac surgery with ECC (coronary, valvular or mixed surgery) (ECC group) and 11 patients with severe sepsis or septic shock (septic group) were included. The CD64 expression on PMNs was quantified by the hematologic Cell Dyn Sapphire method (Abotte US) before T0, at ICU admission (T1) and postoperatively on day 1 (T2) and day 5 (T3) for the ECC group and on days 0, 1 and 5 for the septic group. Values are expressed as median (25th to 75th) percentiles

## Results

Fifty patients were included among which 39 in the ECC group (nine valvular, 20 coronary artery bypass grafting and 10 mixed surgery). As expected, the inflammatory parameters were significantly increased in septic patients compared to the ECC group except on day 5 (for example, CRP: 0.2 (0.1 to 0.6) vs. 12.5 (5.7 to 26.9) mg/dl; WBC 6.5 (5.2 to 8.7) vs. 19.5 (12 to 20.5) 10^3^/mm^3^; for respectively ECC and septic group at T0, *P *< 0.001). The CD64 expression increased significantly in both groups but index values were lower in the ECC compared to the septic group except on T3 (Table 1).

**Table 1:** 

	ECC	Sepsis	*P *value
T0	0.8 (0.6 to 1.08)	3.24 (1.9 to 7.8)	<0.001
T1	0.9 (0.6 to 1.14)^†^	Not available	
T2	1.3 (0.77 to 1.8)*,**	4.4 (2.63 to 6.7)*	<0.001
T3	1.1 (0.74 to 1.4)^‡^	1.3 (0.74 to 1.4)	0.16

*P < 0.05 T2 vs. T3, **T2 vs. T0, ^†^T2 vs. T1, ^‡^T3 vs. T0. ANOVA tests.

## Conclusion

ECC modifies the inflammatory parameters, including the expression of the CD64 on PMNs but this one presents the best specificity to diagnose an infection. Thus, CD64 expression could be proposed as a promising marker in the early diagnosis of the infection.

## References

[B1] Ioan-Fascinay A Immunity 2002 16 391 402 10.1016/S1074-7613(02)00294-7 11911824

[B2] Nuutila J J Immunol Methods 2007 328 189 200 10.1016/j.jim.2007.09.002 17905303

[B3] Qureshi SS Clin Exp Immunol 2001 125 258 265 10.1046/j.1365-2249.2001.01596.x 11529918 PMC1906134

